# Case report: Blindness associated with *Learedius learedi* trematode infection in a green sea turtle, *Chelonia mydas*, of the northern Red Sea

**DOI:** 10.3389/fvets.2023.1258522

**Published:** 2023-09-29

**Authors:** Danny Morick, Vanessa M. Bachmann, Eli Shemesh, Ana Maria Botero-Anug, Ziv Zemach-Shamir, Zahi Aizenberg, Nadav Davidovich, Daphne W. Goldberg, Gaston Kan, Ron Ofri, Dan Tchernov, Yaniv Levy

**Affiliations:** ^1^Morris Kahn Marine Research Station, University of Haifa, Haifa, Israel; ^2^Department of Marine Biology, Leon H. Charney School of Marine Sciences, University of Haifa, Haifa, Israel; ^3^Hong Kong Branch of Southern Marine Science and Engineering, Guangdong Laboratory (Guangzhou), Guangzhou, China; ^4^Pathovet Diagnostic Veterinary Pathology Services, Rehovot, Israel; ^5^Koret School of Veterinary Medicine, Hebrew University of Jerusalem, Rehovot, Israel; ^6^Israeli Veterinary Services, Bet Dagan, Israel; ^7^Instituto Albatroz, Rio de Janeiro, Brazil; ^8^Israeli National Nature and Parks Authority-Israel Sea Turtle Rescue Center, Jerusalem, Israel

**Keywords:** Spirorchiid, *Learedius learedi*, green sea turtle, blindness, optic nerve, Red Sea

## Abstract

Spirorchiid blood flukes are widespread in sea turtles, causing disease and mortality in their populations, with high prevalence in several ocean basins. Besides being leading parasitic causes of sea turtle strandings in several parts of the world, these infectious agents can cause endocarditis, vasculitis, thrombosis, miliary egg granulomas, and aneurysms, which ultimately may compromise the survival of green sea turtles. More severe cases may also result in multifocal granulomatous meningitis or pneumonia, both of which can be fatal. Herein, we report the first case of severe trematode infection, Caused by *Learedius learedi*, in a green sea turtle in the northern Red Sea; this infection is associated with bilateral blindness. Necropsy revealed multiple granulomas with intralesional trematode eggs in the optic nerve, eyes, spleen, heart, and lungs. The parasite was identified as *Learedius learedi* through specific primers of the ribosomal genome and COI sequences obtained from GenBank. Altogether, these findings emphasize the importance of recognizing the systemic nature of this particular fluke infection to ultimately protect the lives of these marine animals and ensure the sustainability of these species in the wild.

## 1. Introduction

Blood trematodes of the Spirorchiidae family affect the cardiovascular systems of freshwater and sea turtles (1, 2). The adult parasites infect the heart and blood vessels, where they oviposit their eggs that migrate to remote areas through the bloodstream, infecting multiple organs and triggering inflammatory responses characterized by disseminated granulomas, arteritis, thrombosis, and aneurysms in major vessels (3, 4). Sea turtles are known to be the definitive hosts of Spirorchiid flukes (5), while intermediate hosts may be polychaete annelids (6) and gastropods, though this cycle has yet to be studied in detail (4). The threat posed by this parasitosis has become a cause for increased conservation concerns, as it may endanger sea turtles.

Ten genera of spirorchiids and its 29 species are known to infect sea turtles (7, 8). The infection is caused by cercariae that penetrate the mucosal membranes. The adults of these spirorchiids lay their eggs in the heart chambers and distal aorta, as revealed by gross examination (9). The eggs are released into the bloodstream and find their way into distant organs of the host, where they may cause a severe inflammatory response in the form of granulomas (10). It is believed that sea turtles shed spirorchiid eggs into the water through their feces (11), although other shedding pathways are suggested, such as respiratory expectoration (8). Further studies are needed to fully understand the routes of egg elimination (8).

Often, the gross lesions go unnoticed or are registered as incidental findings (10). The diagnosis is based on observing adult flukes and gross lesions during the postmortem examination (4). To date, the standard way to diagnose these parasites in live animals is based on the morphology of the eggs by copro-microscopy examination (10). However, in 2020, Marchiori et al. demonstrated the need for more accurate methodologies and consequently developed a blood sample analysis method that provides improved precision (12).

Several species of sea turtles have been affected in various ocean basins. Severe fluke lesions have been reported in Florida (13, 14), Hawaii (15), Costa Rica (16, 17), Brazil (18, 19), and Italy (12, 20), with Australia experiencing the highest prevalence and greatest mortality rates (21–23). This study reports the first case of *Learedius learedi* infection associated with bilateral blindness in a green sea turtle of the Northern Red Sea.

## 2. Case description

### 2.1. Clinical history

**Figure 1:**
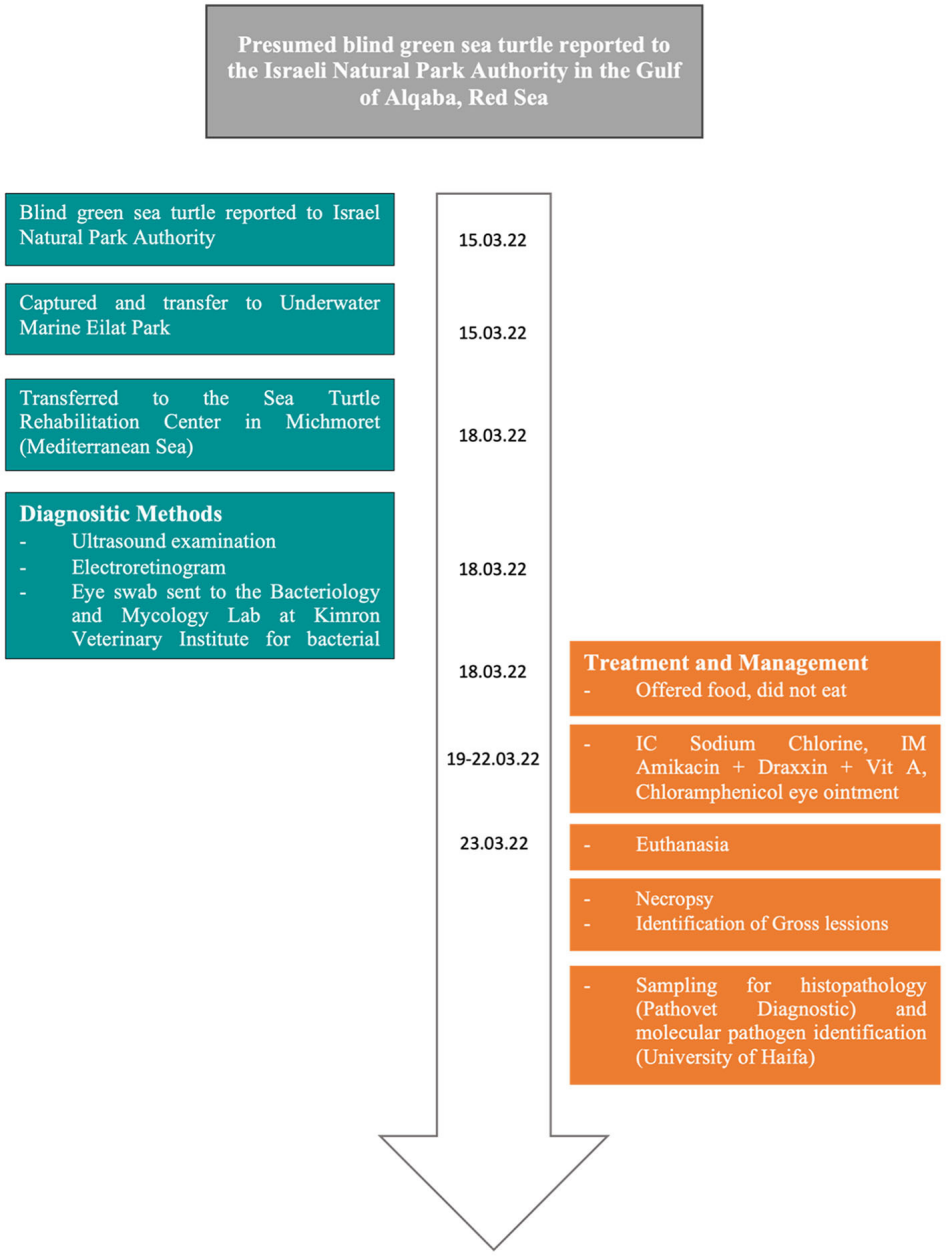
Timeline of events.

On 15 of March 2022, an adult female green sea turtle (*Chelonia mydas*) was reported to the Israeli Nature and Parks Authority (NPA) due to presumed blindness. The animal was spotted by a diver, bumping into the coral reefs at a depth of six m, 20 m off the shoreline in the Gulf of Aqaba (29° 32′ N and 34° 57′ E). Consequently, the specimen was captured by personnel from the Underwater Observatory Marine Park of Eilat and taken into their facility. After 3 days, the sea turtle was transferred to the Sea Turtle Rehabilitation Center located in Michmoret (Mediterranean Sea). Upon admission, the animal was measured (curved carapace length of 68 cm and curved carapace width of 59.5 cm) and weighed (30.26 kg; Figure 1).

### 2.2. Health assessment

**Figure 2:**
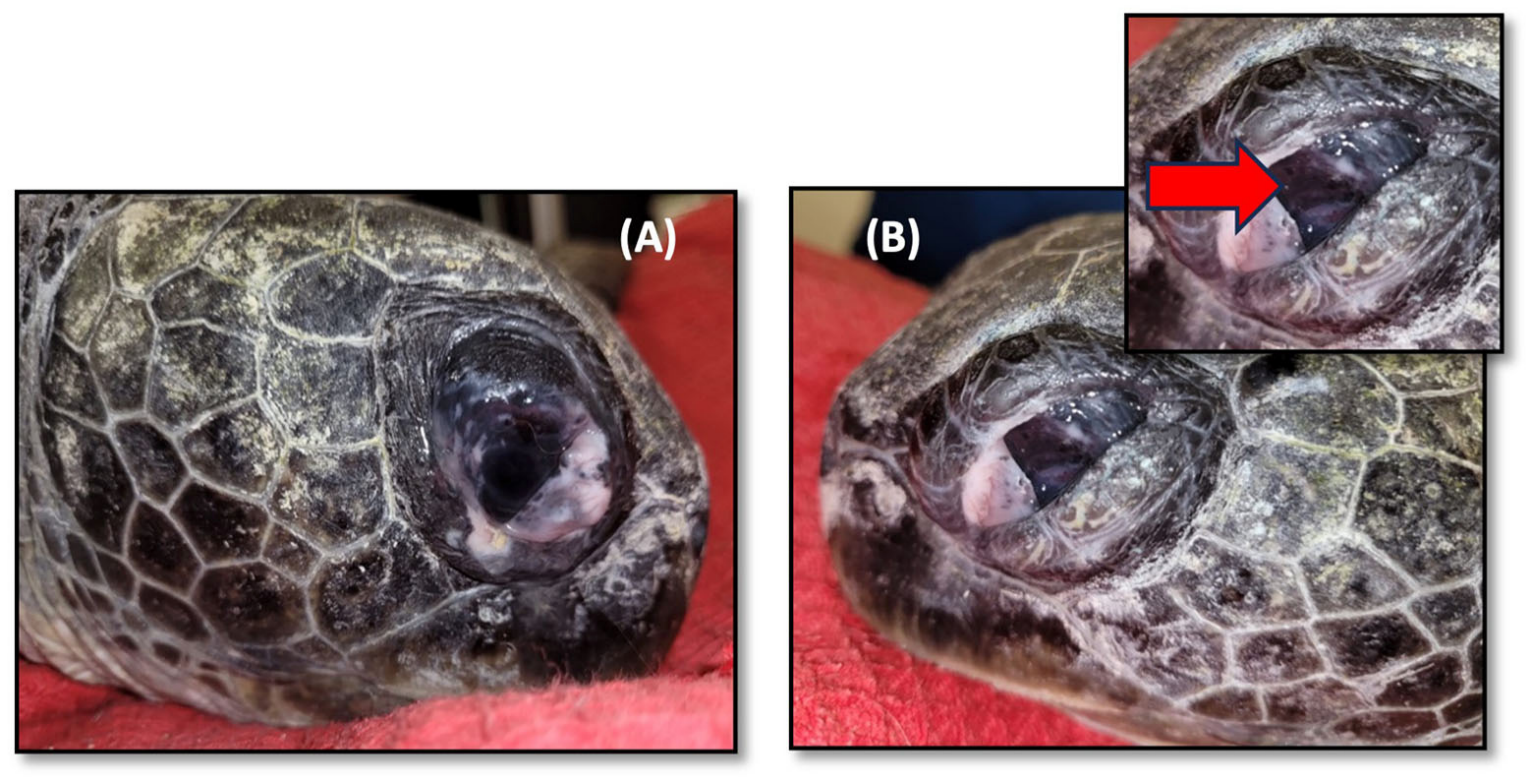
Green sea turtle with *phthisis bulbi* (complete degeneration) due to chronic inflammation caused by *Learedius learedi* infection. **(A)** Right eye, **(B)** left eye. The red arrow shows a shrunken and opaque cornea and thickened sclera.

A physical examination showed bilateral *phthisis bulbi*, which is an end-stage ocular disorder characterized by the shrinkage and disorganization of the eye, ultimately leading to the malfunction of the affected organ (24) (Figure 2). An ultrasound examination revealed no evidence of retinal detachment in either eye. The corneal tissue was observed to be hyperechoic with dystrophy and fibrosis. An electroretinogram (ERG) revealed a lack of retinal function in the right eye. Sample swabs from the eyes were sent to the Bacteriology and Mycology Lab at Kimron Veterinary Institute to assess for the presence of aerobic bacteria and Mycoplasma, with ultimately negative results (Figure 1).

### 2.3. Treatment

The animal was not eating properly, so it was hydrated with 400 ml of 0.9% sodium chloride in an intracoelomic (IC) solution over the course of four days. Additionally, it was treated with amikacin (5 mg/kg q. 48 h) IM, Draxxin^®^ (tulathromycin 2.5 mg/kg) IM, and vitamin A 5000 IU IM. Chloramphenicol 5% eye ointment (Synthomycine^®^) was administered to both eyes to prevent secondary infection (Figure 1).

### 2.4. Euthanasia and postmortem examination

Due to its deterioration and bilateral blindness, the turtle was euthanized using the criteria published by NSW Wildlife Council (25), that state that blind turtles are unable to be released into the wild as they are unable to find food and evade predators, thus rendering them unable to survive. A veterinarian, working with the Nature and Parks Authority, carried out euthanasia in accordance with the NPA's guidelines and Protocol License 891. The protocol used propofol IV (Rapinovet, Schering-Plough Animal Health, USA; 5 m/kg) to anesthetize the animal (26), followed by sodium pentobarbital IV (Pentoject, Animalcare, UK; 80 mg/kg) (27). The death of the animal was confirmed through the observation of rigor mortis. The necropsy was conducted using the Flint (4) protocol. Samples from the eyes, optic nerves, heart, spleen, lungs, and liver were collected for histopathologic examination. These samples were fixed in 10% neutral phosphate-buffered formalin and processed into paraffin blocks for cutting and staining. The blocks were cut into 5 μm, which were then stained with hematoxylin and eosin (H&E).

### 2.5. Histopathologic findings

**Figure 3:**
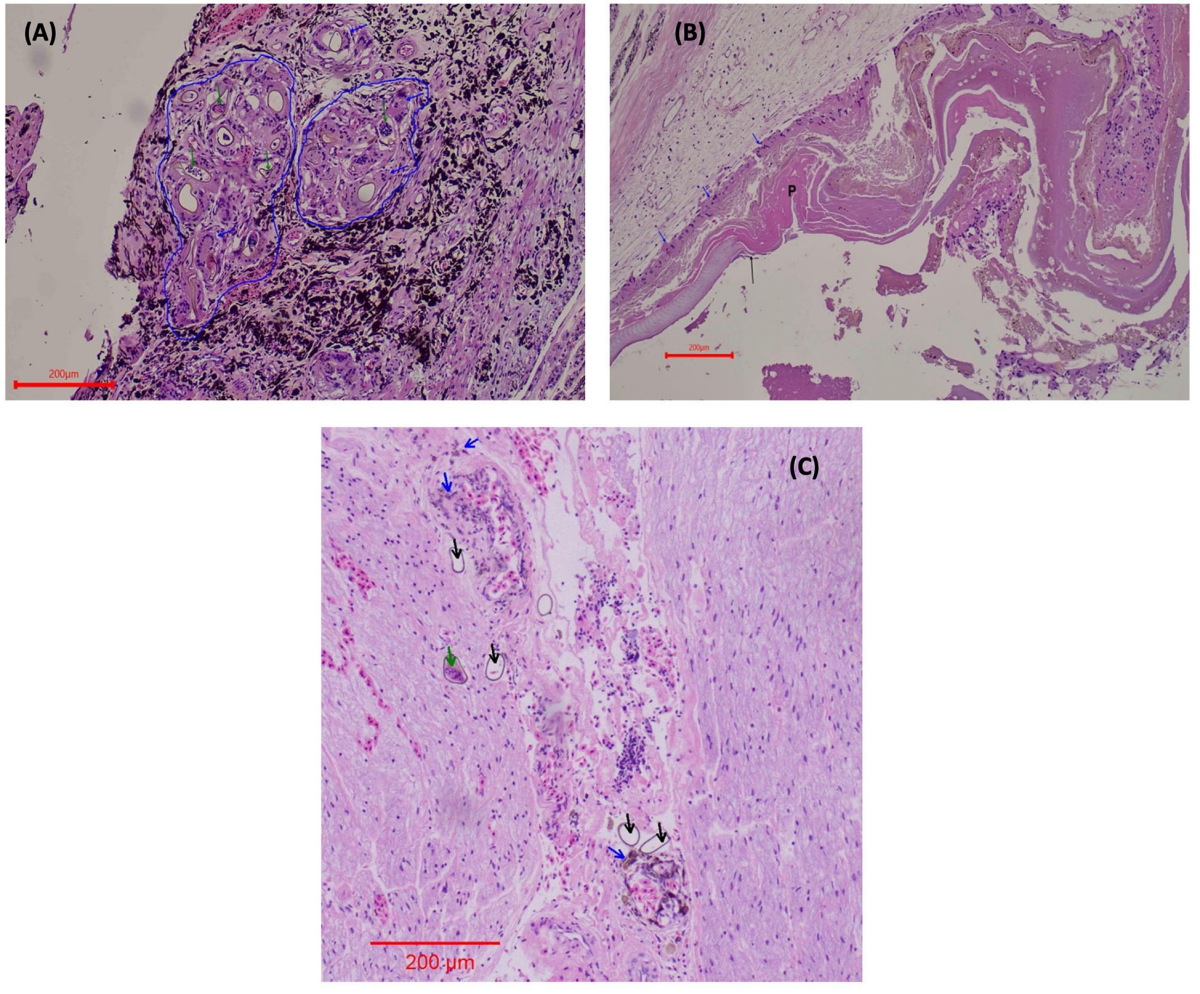
Ocular lesions caused by *L. learedi* eggs in *Chelonia mydas* HE stain. **(A)** Ciliary body of the eye 10X. The blue arrows indicate giant cells surrounding the fluke eggs. The blue line demarcates the coalescing granulomas. The green arrows indicate the embryonated fluke eggs. **(B)** Rupture of the lens of the eye 10X. The blue arrows indicate giant cells (in phagocytosis of protein), P shows the protein from the ruptured lens, and the black arrows are evidence of the ruptured lens (fibers). **(C)** Optic nerve (10X). Green arrows indicate the embryonated fluke eggs; black arrows show the fluke eggs (these eggs are not surrounded by macrophages), and the blue arrows indicate macrophages in the phagocytosis of hemosiderin in perivascular areas.

**Figure 4:**
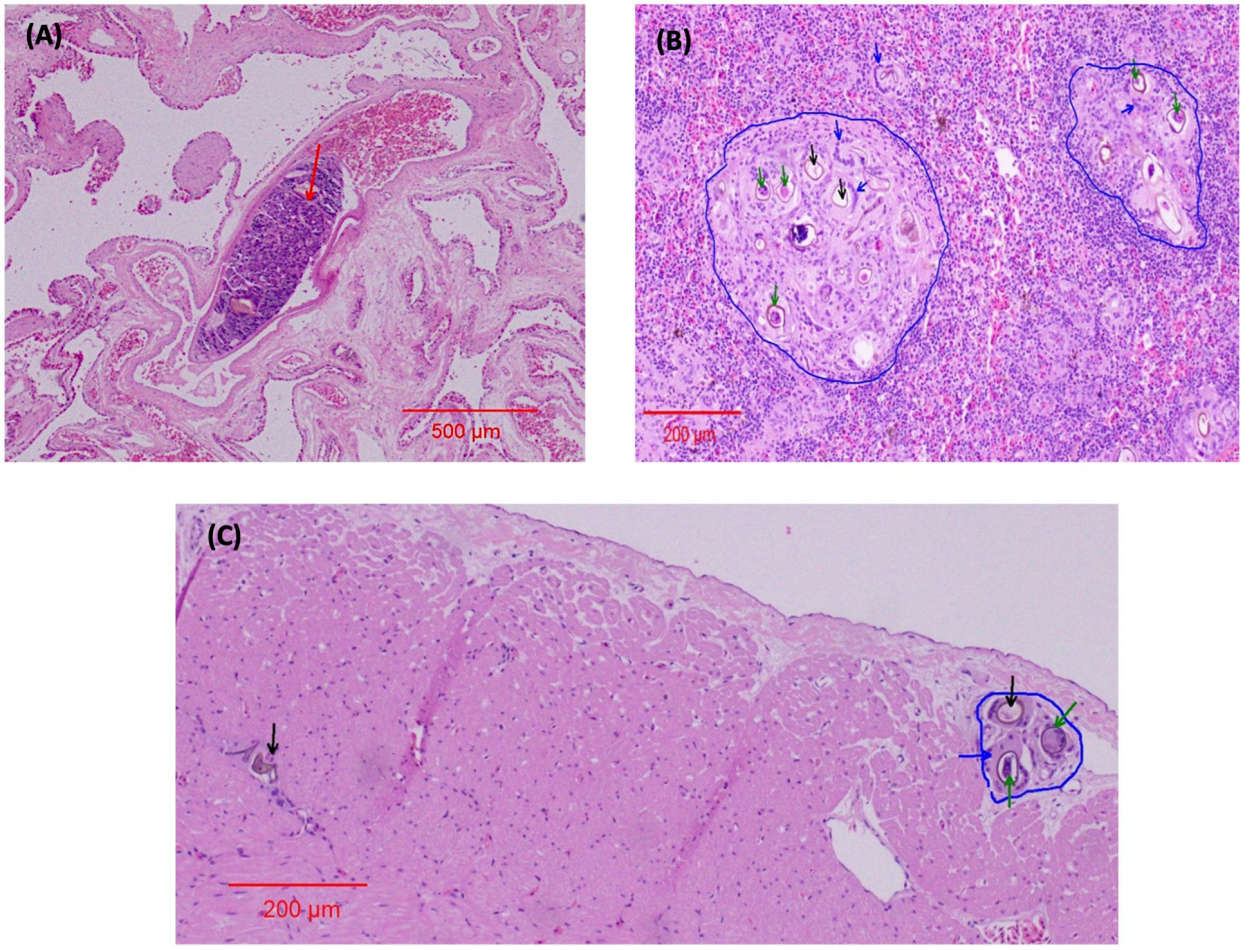
Organ lesions caused by spirorchiid trematodes in *Chelonia mydas* HE stain. **(A)** Lungs 4x. The red arrow indicates the adult fluke in a blood vessel. **(B)** Spleen 10x. The blue arrows indicate giant cells surrounding the fluke eggs; the blue line demarcates the granulomas; the green arrows indicate the embryonated fluke eggs; and the black arrows indicate the fluke eggs. **(C)** Myocardium 10x. The blue arrow indicates a giant cell enveloping the fluke eggs; the blue line demarcates the granuloma; the green arrows indicate the embryonated fluke eggs; and the black arrow indicates the fluke eggs.

In both eyes, mainly between the scleral cartilage and the choroid of the posterior uvea, triangular eggs were observed, evidencing a separation of these two structures. The eggs were surrounded by macrophages and numerous multinucleated giant cells, forming granulomas scattered along the ciliary body and the margin of the eye (Figure 3A). In the left eye, there was an apparent rupture of the lens with abundant proteinaceous material surrounded by multinucleated giant cells (Figure 3B). The determination of the retinal detachment was precluded due to the extension of autolysis of the eye structures. Clusters of eggs surrounded by multinucleated giant cells and numerous capillaries were interspersed within the nerve tissue of one evaluated optic nerve (Figure 3C). The ediculae of the lungs were dilated and clear of contents. Lodged in the lumen of a large pulmonary vein was a large adult trematode (fluke), which was approximately 1 mm long and 0.3 mm wide (Figure 4A). A small, organized luminal thrombus was observed in the second septal vein of the lungs. Multiple granulomas containing trematode eggs were scattered in the spleen (Figure 4B). Fewer granulomas were observed in the myocardium (Figure 4C) and in the interpedicular septae of the lungs.

### 2.6. Molecular identification of the parasite

The total genomic DNA of the sea turtle tissue was extracted using the Wizard SV Genomic DNA Purification System kit (Promega, Madison, WI, USA) following the manufacturer's protocol instructions. A partial sequence of the mitochondrial cytochrome c oxidase I (COI) and 28s (rRNA) genes were amplified using newly designed primers: Trematoda COIF forward primer (5′- ATG GTT CCT AGA ATT TTT TAT ATG GA−3′) and Trematoda COIR reverse primer (3′- ACC CAT AGG ATC AAA AGC AGT ACC−5′). For the 28S (rRNA) fragment, we designed two sets of primers: one for the first PCR, Trematode 28sF1 forward primer (5′- GTC TTG TTC AGT GGG CGG TTG CGT GTG−3′) and Trematoda 28sR1 reverse primer (3′- ACG ATC GAT TTG CAC GTC AGA ATC GC−5′), and a second set for nested PCR, Trematoda 28sF2 forward primer (5′- TTG GGC CAA TAG TCT GTG TAG TGG−3′) and Trematoda 28sR2 reverse primer (3′- GCA TAG TTC ACC ATC TTT CGG GTC TCA A−5′). This step was taken to eliminate turtle sequences from the first PCR, thereby obtaining a more accurate trematode sequence. We performed the PCR analysis in 30 μl volumes with the PCRBIO HS Taq Mix Red enzyme and buffer system (PCR Biosystems Ltd., UK). The COI PCR was performed by a conventional program with an annealing temperature of 50°C. The 28S fragment was evaluated by nested PCR, with an annealing temperature of 60°C at both steps. Both fragments demonstrated 100% homology to other *Learedius learedi* sequences and were deposited in GenBank under the new accession numbers OP800232 and OP800228.

## 3. Discussion

Green sea turtles face population threats across their distribution range, largely due to anthropogenic disturbances (28). In addition, diseases, both naturally occurring and human-induced, may play an important role in their decline. Unfortunately, our understanding of most of these diseases is lacking, and their effects on turtles' health and distribution remain unexplored. In several regions of the world, Spirorchiidiasis blood fluke infection is the leading parasitic cause of morbidity and mortality among green sea turtles (8). This disease has a chronic course and debilitates its host progressively when the ova spreads to numerous organs, exacerbating a granulomatous response (17) that consequently causes multiorgan failure. The present study describes granulomatous lesions in a green sea turtle, resulting from *L. learedi* ova infestation in the eyes, optic nerves, myocardium, and spleen, along with the presence of an adult in the pulmonary vein, as reported by several authors (14, 29–32). The molecular PCR analysis results of our study indicate that the infection was caused by *Learedius learedi*. The 28S sequence (accession number: OP800228) demonstrated 100% homology to other *Learedius learedi* sequences. The COI result (accession number: OP800232) also identified the infection as *Learedius learedi*, albeit with a similarity of 93%. This indicates that the cause of blindness in the green sea turtle was most likely attributed to the presence of *L. learedi* eggs in the optic nerve, choroid, and ciliary body, leading to retinal detachment and evidence of lens rupture in at least one of the eyes.

Stranded green turtles from the Queensland coast in Australia have been affected by blood flukes from the *Hapalotrema, Learedius*, and *Amphiorchis* genera (22). These parasites have caused a great deal of distress among the stranded turtles, leading to their inability to thrive in their natural environment. The infection of *L. learedi* ova in those animals was observed in the brain, heart, gastrointestinal tract, pancreas, spleen, liver, gall bladder, and kidneys (22) but not in the eyes or optic nerves, as observed in this study. Despite causing significant clinical issues in most afflicted turtles, Santoro et al. (16) revealed the presence of the same parasites in healthy female green turtles from Costa Rica. This suggests that the mere presence of these parasites alone is not necessarily indicative of illness.

Ocular spirorchiid trematode infection in sea turtles is a relatively unknown condition, with two regions reporting this matter: Queensland, Australia (8) and the Southwestern Atlantic (29). In the latter location, Jerdy et al. (29) determined that 90% of sea turtles were infected with this parasitic disease and described an egg-induced giant cell inflammatory reaction in the choroid layer of the affected eyes. The choroid layer is composed of blood vessels that supply oxygen and essential nutrients to the outer retina, pigment epithelium, and photoreceptors (29). In cases of severe ocular spirorchiidiosis, however, this layer loses its ability to provide such vital nutrients and instead releases inflammatory factors that can damage the cells and structures of the retina (33). Less severe cases could cause focal visual loss due to the thickening of the choroid layer (29). Jerdy et al. (29) found neuritis in the optic nerves (*n* = 30) caused by spirorchiid eggs, a condition that affects the signal transmission of the nerves (32). Glazebrook et al. also found ocular lesions in green sea turtles but without any description of the extent of the choroid layer (32). Based on the research conducted, it is likely that ocular spirorchiidosis will disrupt eye structure and cause blindness, and further research should be conducted to better understand the extent and effect of this parasitic infection (29).

An association has been found between the severity of ocular spirorchiidiosis and the body condition of sea turtles, with a three-fold probability of cachexia in severely infected animals compared to healthy ones (29). In contrast, the turtle in this study presented good body condition despite being blind, suggesting that it had only recently developed bilateral blindness. Due to its blindness, its ability to forage would soon be compromised. Guidelines for the treatment and care of rescued sea turtles identified specific morbidities that are incompatible with survival in the wild (25), including bilateral blindness. In fact, spirorchiidiasis may have a significant effect on the animal's capability for foraging, jeopardizing its chance of survival in the wild (25).

For live animals, copro-microscopy is typically used as the diagnostic method for detecting spirorchiid infections (8). However, research has shown that the correlation between fecal and splenic egg counts in loggerhead sea turtles is relatively poor, meaning that this method is not suitable for accurately quantifying their parasite burden (12). Chapman et al. (34) developed a fluorescence-based PCR T-RFLP method (terminal restriction fragment length polymorphism) to identify spirorchiid eggs in tissue samples of green turtles. Furthermore, real-time PCR has opened up a range of possibilities for schistosomiasis diagnosis and post-treatment monitoring across various species. It has provided a way to quickly diagnose the presence of the disease by using fecal samples, as well as a means of tracking the progress of treatment response. These insights have the potential to greatly improve diagnosis and treatment of blood fluke in sea turtles (8). Early detection is critical to avoiding severe infestation and blindness, which can both significantly compromise the animal's survival in the wild. Praziquantel, an anthelminthic, is recommended as a treatment for spirorchiidiasis, with a dose of 25 mg/kg administered orally in three separate doses at 3-h intervals over the course of 1 day (35). Unfortunately, there is a poor response in cases of extensive egg embolization (35).

Spirorchiidiosis is a potentially serious, yet often overlooked, cause of morbidity and mortality in sea turtles. Without prompt diagnosis and proper care, the diseases caused by spirorchiid infections can have detrimental effects on a turtle's health and lead to premature death. It is, therefore, of utmost importance that stranded turtles be routinely assessed for spirorchiid infections so that they can receive the medical treatments needed to aid in their recovery. Through the prompt and accurate diagnosis of spirorchiidiosis, veterinarians and wildlife rehabilitators can ensure that sea turtles receive the necessary medical care and improve their chances of survival.

## Data availability statement

The datasets presented in this study can be found in online repositories. The names of the repository/repositories and accession number(s) can be found at: https://www.ncbi.nlm.nih.gov/genbank/, OP800232; https://www.ncbi.nlm.nih.gov/genbank/, OP800228.

## Ethics statement

Ethical approval was not required for the study involving animals in accordance with the local legislation and institutional requirements because the treatment and management of the animals are allowed by the Nature and Parks Authority according to the guidelines and protocol license 891.

## Author contributions

DM: Data curation, Formal analysis, Funding acquisition, Investigation, Writing—original draft, Writing—review and editing. VB: Formal analysis, Writing—original draft, Writing—review and editing. ES: Formal analysis, Writing—review and editing. AB-A: Formal analysis, Writing—review and editing. ZZ-S: Formal analysis, Writing—review and editing. ZA: Data curation, Formal analysis, Writing—review and editing. ND: Writing—review and editing. DG: Formal analysis, Writing—review and editing. GK: Data curation, Formal Analysis, Writing—review and editing. RO: Data curation, Formal analysis, Investigation, Writing—original draft, Writing—review and editing. DT: Conceptualization, Writing—review and editing. YL: Conceptualization, Data curation, Formal analysis, Writing—review and editing.
